# The Effectiveness of Peer Group-Based Training on the Outcomes of Patients Undergoing Transradial Coronary Angiography

**DOI:** 10.1155/2020/3629782

**Published:** 2020-04-26

**Authors:** Ahmad Reza Dehghan, Zhila Fereidouni, Majid Najafi Kalyani

**Affiliations:** ^1^Fasa University of Medical Sciences, Fasa, Iran; ^2^School of Nursing and Midwifery, Shiraz University of Medical Sciences, Shiraz, Iran

## Abstract

**Introduction:**

Coronary artery angiography using radial artery is one of the methods used for diagnosis of coronary artery disease, which causes physical and psychological problems in patients despite its precise and definite diagnosis. The present study is aimed at investigating the effect of peer group-based education on physical and psychological outcomes of patients undergoing coronary artery angiography through the radial artery. *Methodology*. The present clinical study was conducted on 60 patients undergoing coronary angiography through the radial artery in Vali-e-Asr educational hospital of Fasa during 2018 to 2019. The participants were divided into peer training and control groups (*n* = 30 in each group) using permutated block randomization. In the peer training group, the patients received the necessary precare training through peer training during and after angiography care. In the control group, the patients received the routine care by the nurse of the related ward. The peer group's stress, anxiety, and depression levels were evaluated before and after the training. Indeed, their comfort, tolerance, satisfaction, and pain levels were measured by a nurse after angiography at the time of entering the ward. *Findings*. The results indicated no significant difference between the two groups regarding the mean scores of stress, anxiety, and depression before the intervention (*p* > 0.05). After the intervention, however, there was a significant difference between the two groups concerning the mean score of anxiety (*p* < 0.05). Nonetheless, no significant difference was found between the two groups in terms of tolerance, comfort, satisfaction, and pain levels (*p* > 0.05). Finally, the level of pain decreased in both groups over time (*p* < 0.001).

**Conclusion:**

Peer group-based training was effective in decreasing the mean score of anxiety in the patients undergoing coronary angiography. Thus, this method is recommended to be utilized alongside other methods to train patients before coronary angiography due to its inexpensiveness and lack of side effects as well as not increasing the nurses' workload.

## 1. Introduction

Coronary artery angiography is one of the most accurate diagnostic methods for coronary artery disease, in which the size and percentage of coronary artery stenosis are specified [1, 2]. The use of this diagnostic test has increased in the recent years due to the increased number of people with coronary artery disease [3]. According to statistics, more than a million coronary angiographies occur in the United States annually [1]. Coronary angiography is carried out as a diagnostic test over the last few decades [4].

Coronary angiography causes several problems in patients undergoing this procedure due to its invasive nature despite its high potential for accurate diagnosis of coronary artery disease [5, 6]. The conducted studies in this field have shown that this diagnostic test was stressful for many patients and caused fear and anxiety [7, 8]. Indeed, the occurrence of psychological problems in patients undergoing this procedure could increase the workload of the heart and cause such problems as chest pain, cardiac arrhythmia, and heart attack [9]. Coronary angiography could also lead to physical problems, such as hemorrhage, hematoma, pain, nausea, and vomiting in addition to psychological problems [5, 9, 10]. Evidence has shown that patients experienced psychological problems due to lack of awareness, fear from unknowns, and lack of control over themselves and the environment [7, 11].

Various pharmaceutical and nonpharmaceutical methods have been used to manage the psychological and physical problems of the patients undergoing coronary angiography [12, 13]. Nonpharmacological interventions have been increasingly used due to cheapness, lack of known side effects, and better acceptance by patients [13]. Training the patients is one of the nonpharmacological methods, which can be carried out through a variety of ways [14]. Considering the increasing number of patients and shortage of nurses, peer group training method has been suggested as one of the methods that can be used for training the patients [15]. This method has been more widely accepted by the patients because the trainer has the experience of dealing with the problem and transfers one's thoughts and feelings to the patients [16, 17].

Up to now, various studies have been conducted on the effect of training interventions on the patients' problems under coronary angiography through femoral arteries [8, 14]. As transradial coronary angiography is a new approach in Iran, few studies have been performed on utilization of training interventions to manage the problems of the patients under coronary artery angiography through the radial artery. On the other hand, most of the studies have only put emphasis on either physical or psychological problems [8, 12, 14, 18]. Considering the importance of managing physical and psychological problems in the patients undergoing coronary artery angiography as well as the necessity to make use of nonpharmacological and low-cost methods to manage these problems, the present study is aimed at investigating the effect of peer group-based education on physical and psychological outcomes of the patients undergoing coronary artery angiography through the radial artery.

## 2. Methods

### 2.1. Study Design

The present clinical study was conducted on 60 patients undergoing coronary angiography through the radial artery in Vali-e-Asr educational hospital of Fasa during August 2018 to February 2019.

### 2.2. Participants

In the present study, 60 patients were selected through simple random sampling by random number table and were entered into the study after investigation of the inclusion and exclusion criteria. The inclusion criteria were undergoing transradial coronary angiography, undergoing coronary angiography for the first time, aging 30-70 years, having no cognitive, behavioral, and verbal disorders, having the ability to speak, having the physical ability to attend the training session, and being willing to cooperate. The exclusion criteria were coadministration of coronary angioplasty and having the history of receiving antianxiety and antidepression drugs.

**Figure 1:**
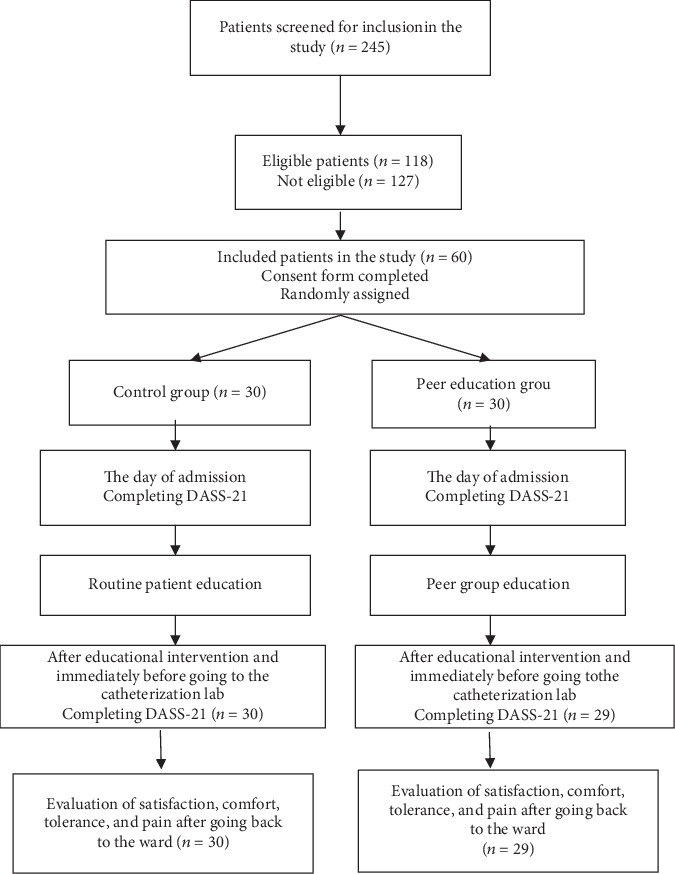
The CONSORT flow diagram of the stages of the clinical study.

According to the study by Jamshidi et al. [18] and considering stress score as the initial outcome, alpha = 0.05, and power = 80%, a 56-subject sample size was estimated for the study. Yet, the sample size was increased to 60 (*n* = 30 in each group) by considering the probability of loss (Figure 1).

### 2.3. Randomization

At first, the patients were provided with complete explanation about the research methods and were required to sign written informed consent forms for taking part in the study. Then, they were randomly divided into peer training and control groups based on permuted block randomization (AB and BA blocks) using a random number generator. The patients were allocated to the study groups every other day by coin throw method to prevent them from communicating with each other. Therefore, the patients in the two study groups had no interactions with each other. The study process has been depicted in Figure 1.

### 2.4. Intervention

The patients in the peer-based group received the training three hours prior to coronary artery angiography. The training in the educational session included the necessary preparations before angiography, care during angiography, postangiography care, and the measures that should be taken by the patients after discharge. The peer group training lasted for 45 minutes, and the training was carried out via lecture, group discussion, role play, and question and answer. The training was conducted by two peers in groups of three to four patients in the training room of the hospitalization unit. The peers were selected by such criteria as having at least a diploma degree, passage of at least three months from their angiography, and willingness to train other patients. The peers were trained in the hospital by the researchers through lecture, question and answer, group discussion, and role play one week before sampling. Indeed, the trained topics were discussed at the end of the training sessions in order to ensure the peers' understanding. In the control group, the patients were provided with the routine care and training during admission by the nurse of the angiography unit. The training included the need for fasting, hand shaving, and how to take medications.

### 2.5. Measurement

After dividing the participants into peer training and control groups, they were asked to complete the demographic questionnaire and Depression, Anxiety, Stress Scale-21 (DASS-21) (pretest). After the training and immediately before going to the catheterization lab, the patients were again asked to complete DASS-21 (posttest).

DASS-21 has been used in several studies and its validity and reliability have been confirmed [18, 19]. This questionnaire is a set of three self-report scales (from 0 to 3) designed to measure depression, anxiety, and stress. Each of the three DASS-21 subscales contains seven items divided into subscales with the similar content.

Immediately after the end of angiography and transferring the patients from the catheterization laboratory to the hospitalization unit, the patients' pain was measured by a nurse using a Visual Analogue Scale (VAS) in five stages, i.e., at the time of arrival, the first hour, the second hour, the fourth hour, and the fifth hour. The scores of this scale range from 0 to 10, representing no pain and the most severe pain, respectively. Visual comparative measures have been used in several studies to measure mental variables [9, 20].

The patients' satisfaction, tolerance, and comfort were measured by a nurse five hours after their entrance into the hospitalization unit. The patients' tolerance was evaluated using a four-point scale with the following options: completely tolerable, relatively tolerable, relatively intolerable, and completely intolerable. Additionally, satisfaction was assessed via a five-point Likert scale, which included totally satisfied, relatively satisfied, no idea, relatively dissatisfied, and completely dissatisfied. These scales were used in the previous studies to measure the patients' tolerance [21] and satisfaction [22]. Finally, the patients' comfort was measured by a nurse using a VAS five hours after being transferred to the hospitalization unit. This scale ranged from 0 to 10, representing severe discomfort and complete comfort, respectively. This tool has been used in several studies to measure the comfort of the patients under coronary angiography [9].

### 2.6. Ethical Considerations

The present study was approved by the Ethics Committee of Fasa University of Medical Sciences (IR.FUMS.REC.1397.024). Written consent forms were obtained from the patients after providing them with explanation about the research objectives and methodology. In addition, the patients were assured that they could withdraw from the study whenever they did not want to cooperate.

### 2.7. Statistical Analysis

Data analysis was carried out using the SPSS statistical software, version 22. Chi-square, Fisher's exact test, and independent samples *t*-test were used to compare the two groups in terms of the outcome variables. *p* < 0.05 was considered to be statistically significant.

## 3. Results

In the present study, one patient was excluded due to the incompleteness of the questionnaires. Thus, a total of 59 patients were entered into the final analysis. According to Table 1, there was no statistically significant difference between the two groups in terms of age, body mass index (BMI), gender, occupation, and marital status.

The results showed no significant differences between the two groups regarding the mean scores of stress, anxiety, and depression before the intervention (*p* > 0.05) (Table 2). However, the results of *t*-test revealed a significant difference between the two groups concerning the mean score of anxiety after the intervention (*p* < 0.05). Nonetheless, no statistically significant difference was observed between the two groups with respect to the mean scores of stress and depression after the intervention (*p* > 0.05) (Table 2).

The results of paired *t*-test indicated a significant difference between the peer training group's mean scores of anxiety before and after the intervention (*p* < 0.05). Accordingly, the mean score of anxiety decreased after the intervention compared to the baseline. In the control group also, there was a significant difference between the mean scores of anxiety before and after the intervention, but the difference followed an increasing trend compared to before the intervention (*p* < 0.05). The use of peer group causes a nonsignificant decrease in the stress score of patients compared with the control group (Table 3).

The results of Fisher's exact test revealed no significant difference between the two groups in terms of satisfaction and tolerance levels. In addition, the results of *t*-test indicated no significant difference between the two groups with regard to the mean scores of pain and comfort (Table 4). The trend of the patients' mean scores of pain in the two groups was investigated using repeated measures ANOVA. The results demonstrated a significant difference between the trends of the patients' mean scores of pain in the two groups over time (*p* < 0.05). According to Table 4, the pain level followed a descending trend in the peer training group over time compared to the control group.

## 4. Discussion

The results of the present study showed that peer group-based training significantly decreased the mean score of anxiety among the patients under coronary angiography compared to the control group. Consistently, Molazem et al. showed in their study in Iran that peer group training could decrease the anxiety score of the patients under coronary angiography through the femoral artery [8]. Similarly, Habibzadeh et al. in Iran reported that peer group training led to a further decrease in the patients' anxiety scores compared to other training methods [23]. Varaei et al. also indicated that peer group training as a method of patient training decreased the anxiety level [24]. Considering the patients' lack of information and awareness about coronary artery angiography and the resultant anxiety, providing the patients with information on the issue would increase their awareness and decrease their anxiety level [8, 18].

The results of the present study showed that peer group training caused a nonsignificant decrease in the stress score of the patients in the intervention group compared to the control group. In the same vein, the results of a study conducted by Jamshidi et al. in Iran demonstrated that providing the patients undergoing coronary artery angiography with information through displaying a video could significantly decrease their stress levels [18]. In addition, the results obtained by Kumakech et al. indicated that support and training through peers decreased psychological distress [25]. These results were also in agreement with those of the study by Eslami et al., which showed that utilizing peer groups could decrease stress in the patients undergoing coronary angiography [26]. Understanding and respecting the patients' opinions by the peer group could lead to better acceptance of the training and reduction of psychological problems [23].

The findings of the current study showed that peer group training had no significant effects on the depression score among the patients undergoing coronary angiography. The results of the study conducted by Molazem et al. also indicated that peer group training had no significant effects on the depression level of the patients undergoing coronary angiography [8]. Similarly, the studies conducted on the effectiveness of training interventions in coronary artery angiography have shown that these interventions had no significant impacts on the patients' depression scores [18].

In the present study, no significant difference was observed between the two groups in terms of satisfaction, tolerance, and comfort. On the contrary, Jamshidi et al. showed that training through displaying films could lead to more satisfaction, tolerance, and comfort among the patients under coronary angiography compared to the control group [27]. In addition, the results of a study conducted by Sweet et al. showed that peer group training could provide more satisfaction with life among the patients with spinal cord injury [28]. Rezaei-Adaryani et al. also reported more comfort in the patients' position change after coronary artery angiography [9]. The difference between the results of the present study and those of the above-mentioned studies could be related to the type of training as well as the duration of its implementation. In the present study, peer group training lasted for 45 minutes, with more emphasis on the lecture method.

Investigating the patients' pain levels through five stages indicated a decrease in the pain levels in both groups over time. Although this difference was statistically significant within the groups, there was no significant difference between the two groups in this regard, except for the first hour after angiography. However, Rezaei-Adaryani et al. disclosed that position change training decreased the patients' pain [29]. Generally, staying in a fixed position after angiography causes discomfort and pain [20]. The difference between the results of the present study and those of other investigations could be attributed to the type of training as well as duration of implementation. Pain reduction over time can also be attributed to the awareness of patients by education.

One of the most important strengths of the present study was the simultaneous evaluation of the physical and psychological outcomes of the patients under coronary artery angiography through radial arteries, which has not been discussed in the previous studies. Nonetheless, one of the limitations of the study was that although the patients in the two groups had no interactions with each other, the information received from other sources could not be controlled. Indeed, self-report tools were used to evaluate the patients' outcomes. Hence, future studies are recommended to utilize the patients' hemodynamic variables and blood parameters along with self-report tools in order to measure their psychological variables.

## 5. Conclusion

The study findings indicated that peer group-based training could decrease the anxiety and stress scores of the patients under coronary angiography through the radial artery compared to the control group. Therefore, this method is recommended to be utilized for psychological preparation alongside other methods in hospitals of Iran to train patients before coronary angiography due to its inexpensiveness and lack of side effects as well as not increasing the nurses' workload. Future studies are required to evaluate the effects of peer group-based training on managing physical outcomes of patients undergoing coronary angiography.

## Figures and Tables

**Table 1:** Characteristics of the participants.

		Peer education (*n* = 29)	Control (*n* = 30)	*p* value
		Frequency (percent)	Frequency (percent)	
Gender	Male	16 (55.2%)	19 (63.3%)	0.524^∗^
	Female	13 (44.8%)	11 (36.7%)	
Marital status	Married	27 (93.1%)	27 (90.0%)	1.000^∗∗^
	Widowed	2 (6.9%)	3 (10.0%)	
History of hospitalization	Yes	21 (72.4%)	26 (86.7%)	0.209^∗^
	No	8 (27.6%)	4 (13.3%)	
Age		60.68 ± 14.64	64.40 ± 11.35	0.208^∗∗∗^
Body mass index		25.85 ± 3.64	23.88 ± 3.06	0.028^∗∗∗^

^∗^Chi-square test, ^∗∗^Fisher's exact test, and ^∗∗∗^independent samples *t*-test.

**Table 2:** Comparison of the mean scores of anxiety, stress, and depression between the study groups.

Group	Before the intervention			After the intervention		
	Mean ± SD			Mean ± SD		
	Stress	Anxiety	Depression	Stress	Anxiety	Depression
Peer education	7.72 ± 5.04	8.51 ± 3.85	6.55 ± 3.30	6.06 ± 4.30	6.24 ± 3.42	6.93 ± 4.13
Control	6.60 ± 4.02	7.03 ± 3.89	5.13 ± 4.11	7.30 ± 4.30	8.16 ± 3.90	6.10 ± 4.03
*p* value^∗^	0.347	0.147	0.151	0.276	0.049	0.438

^∗^Independent samples *t*-test.

**Table 3:** Comparison of the mean scores of anxiety, stress, and depression within the study groups.

Group	Peer education			Control		
	Mean ± SD			Mean ± SD		
Time	Stress	Anxiety	Depression	Stress	Anxiety	Depression
Before	7.72 ± 5.04	8.51 ± 3.85	6.55 ± 3.30	6.60 ± 4.02	7.03 ± 3.89	5.13 ± 4.11
After	6.06 ± 4.30	6.24 ± 3.42	6.93 ± 4.13	7.30 ± 4.30	8.16 ± 3.90	6.10 ± 4.03
*p* value^∗^	0.069	0.003	0.309	0.055	0.030	0.089

^∗^Paired samples *t*-test.

**Table 4:** Comparison of satisfaction, tolerability, comfort, and pain levels of patients in the study groups.

Group		Peer education (*n* = 29)	Control (*n* = 30)	*p* value
Variable				
Satisfaction	Most satisfied	12 (41.4%)	15 (50.0%)	0.757^∗^
	Satisfied	13 (44.8%)	11 (36.7%)	
	Dissatisfied	4 (13.8%)	3 (10%)	
	Most dissatisfied	0 (0.0%)	1 (3.3%)	

Tolerability	Very tolerable	11 (37.9%)	7 (23.3%)	0.141^∗^
	Tolerable	15 (51.7%)	13 (43.3%)	
	Hardly tolerable	3 (10.3%)	9 (30.9%)	
	Intolerable	0 (0.0%)	1 (3.3%)	

Comfort		6.96 ± 2.74	7.10 ± 2.83	0.854^∗∗^

Pain	Time 0	5.37 ± 2.27	4.93 ± 3.38	0.556^∗∗^
	Time 1	4.51 ± 2.23	3.16 ± 2.47	0.032^∗∗^
	Time 2	4.24 ± 2.45	3.30 ± 2.42	0.144^∗∗^
	Time 3	3.96 ± 2.52	3.20 ± 2.67	0.263^∗∗^
	Time 4	3.51 ± 2.48	2.73 ± 2.39	0.223^∗∗^

*p* value^∗∗^		0.000^∗∗∗^	0.000^∗∗∗^	

^∗^Fisher's exact test, ^∗∗^independent samples *t*-test, and ^∗∗∗^repeated measures ANOVA.

## Data Availability

The data used to support the findings of this study are available from the corresponding author upon request.

## References

[1] Mozaffarian D., Benjamin E. J., Go A. S., on behalf of the American Heart Association Statistics Committee and Stroke Statistics Subcommittee (2016). Heart disease and stroke Statistics—2016 Update. *Circulation*.

[2] Zhang W. J., Yan J. C., Wang Z. Q. (2016). Application of the interventional limb raising management strategy (ILRMS) at radial vascular access sites in coronary angiography and percutaneous coronary intervention: a randomized trial. *International Journal of Nursing Sciences*.

[3] Aviles R. J., Askari A. T., Messerli A. W. (2004). *Introductory Guide to Cardiac Catheterization*.

[4] Kazemian F., Jalali S. F., Hajian-Tilaki K., Arzani A., Amin K. (2018). Underlying risk factors and their relationship with extent of coronary vessel involvement in patients undergoing coronary angiography in north of Iran. *Caspian Journal of Internal Medicine*.

[5] Gallagher R., Trotter R., Donoghue J. (2010). Preprocedural concerns and anxiety assessment in patients undergoing coronary angiography and percutaneous coronary interventions. *European Journal of Cardiovascular Nursing*.

[6] Fereidouni Z., Morandini M. K., Kalyani M. N. (2019). The efficacy of interventions for back pain in patients after transfemoral coronary angiography: a rapid systematic review. *Journal of Vascular Nursing*.

[7] Sharif F., Kalyani M. N., Ahmadi F., Iman M. T. (2018). In the shadow of perceived threat: the live experience of Iranian patients candidate for undergoing coronary angiography. *Journal of Vascular Nursing*.

[8] Molazem Z., Shahabfard Z., Askari A., Kalyani M. N. (2018). Effects of a peer- led group education on fear, anxiety and depression levels of patients undergoing coronary angiography. *Investigación y Educación en Enfermería*.

[9] Rezaei-Adaryani M., Ahmadi F., Asghari-Jafarabadi M. (2009). The effect of changing position and early ambulation after cardiac catheterization on patients’ outcomes: a single-blind randomized controlled trial. *International Journal of Nursing Studies*.

[10] Bogabathina H., Shi R., Singireddy S. (2018). Reduction of vascular complication rates from femoral artery access in contemporary women undergoing cardiac catheterization. *Cardiovascular Revascularization Medicine*.

[11] Kalyani M. N., Sharif F., Ahmadi F., Iman M. T. (2013). Iranian patient’s expectations about coronary angiography: a qualitative study. *Iranian Journal of Nursing and Midwifery Research*.

[12] Goudarzi Y. M., Ghadirian F., Vahedian A., Pishgoo A. (2018). The effect of Benson relaxation on the anxiety of patients under radial angiography: a randomized clinical trial. *Critical Care*.

[13] Carroll D. L., Malecki-Ketchell A., Astin F. (2017). Non-pharmacological interventions to reduce psychological distress in patients undergoing diagnostic cardiac catheterization: a rapid review. *European Journal of Cardiovascular Nursing*.

[14] Ayasrah S. M., Ahmad M. M. (2016). Educational video intervention effects on periprocedural anxiety levels among cardiac catheterization patients: a randomized clinical trial. *Research and Theory for Nursing Practice*.

[15] Gillespie P., O’Shea E., Paul G., O’Dowd T., Smith S. M. (2012). Cost effectiveness of peer support for type 2 diabetes. *International Journal of Technology Assessment in Health Care*.

[16] Varaei S., Shamsizadeh M., Cheraghi M. A., Talebi M., Dehghani A., Abbasi A. (2017). Effects of a peer education on cardiac self-efficacy and readmissions in patients undergoing coronary artery bypass graft surgery: a randomized-controlled trial. *Nursing in critical care.*.

[17] Sharif F., Abshorshori N., Tahmasebi S., Hazrati M., Zare N., Masoumi S. (2010). The effect of peer-led education on the life quality of mastectomy patients referred to breast cancer-clinics in Shiraz, Iran 2009. *Health and Quality of Life Outcomes*.

[18] Jamshidi N., Abbaszadeh A., Kalyani M. N. (2009). Effects of video information on anxiety, stress and depression of patients undergoing coronary angiography. *Pakistan Journal of Medical Sciences*.

[19] Moradipanah F., Mohammadi E., Mohammadil A. (2009). Effect of music on anxiety, stress, and depression levels in patients undergoing coronary angiography. *Eastern Mediterranean Health Journal*.

[20] Chair S. Y., Thompson D. R., Li S. K. (2007). The effect of ambulation after cardiac catheterization on patient outcomes. *Journal of Clinical Nursing*.

[21] Ayral X., Gicquere C., Duhalde A., Boucheny D., Dougados M. (2002). Effects of video information on preoperative anxiety level and tolerability of joint lavage in knee osteoarthritis. *Arthritis Care & Research*.

[22] Astley C. M., Chew D. P., Aylward P. E., Molloy D. A., De Pasquale C. G. (2008). A randomised study of three different informational AIDS prior to coronary angiography, measuring patient recall, satisfaction and anxiety. *Heart, Lung and Circulation*.

[23] Habibzadeh H., Milan Z. D., Radfar M., Alilu L., Cund A. (2018). Effects of peer-facilitated, video-based and combined peer-and-video education on anxiety among patients undergoing coronary angiography: randomised controlled trial. *Sultan Qaboos University Medical Journal*.

[24] Varaei S. H., Cheraghi M. A., Seyedfatemi N., Talebi M., Bahrani N., Dehghani A. (2013). Effect of peer education on anxiety in patients candidated for coronary artery bypass graft surgery: a randomized control trial. *Journal of Nursing Education*.

[25] Kumakech E., Cantor-Graae E., Maling S., Bajunirwe F. (2009). Peer-group support intervention improves the psychosocial well-being of AIDS orphans: cluster randomized trial. *Social Science & Medicine*.

[26] Eslami R., Farsi Z., Azam S. S. (2015). Comparing the effect of peer education and entation tour on the stress of patients candidate for coronary angiography in selected hospital of AJA University of Medical Sciences. *The Journal of Urmia Nursing and Midwifery Faculty*.

[27] Jamshidi N., Abbaszadeh A., Kalyani M. N., Sharif F. (2013). Effectiveness of video information on coronary angiography patients’ outcomes. *Collegian*.

[28] Sweet S. N., Noreau L., Leblond J., Martin Ginis K. A. (2016). Peer support need fulfillment among adults with spinal cord injury: relationships with participation, life satisfaction and individual characteristics. *Disability and Rehabilitation*.

[29] Rezaei-Adaryani M., Ahmadi F., Mohamadi E., Asghari-Jafarabadi M. (2009). The effect of three positioning methods on patient outcomes after cardiac catheterization. *Journal of Advanced Nursing*.

